# Chronic Kidney Disease as a Comorbidity in Heart Failure

**DOI:** 10.3390/ijms24032988

**Published:** 2023-02-03

**Authors:** Magdalena Szlagor, Jill Dybiec, Ewelina Młynarska, Jacek Rysz, Beata Franczyk

**Affiliations:** 1 Department of Nephrocardiology, Medical University of Lodz, ul. Zeromskiego 113, 90-549 Lodz, Poland; 2 Department of Nephrology, Hypertension and Family Medicine, Medical University of Lodz, ul. Żeromskiego 113, 90-549 Łódź, Poland

**Keywords:** heart failure (HF), chronic kidney disease (CKD), GFR, ejection fraction, renin–angiotensin–aldosterone system inhibitors (RAAS-I), mineralocorticoid receptor antagonists (MRAs), sodium–glucose cotransporter 2 inhibitors (SGLT2), kidney replacement, angiotensin receptor–neprilysin inhibitor (ARNI), uremic toxins, finerenone

## Abstract

Heart failure (HF) is one of the greatest problems in healthcare and it often coexists with declining renal function. The pathophysiology between the heart and the kidneys is bidirectional. Common mechanisms leading to the dysfunction of these organs result in a vicious cycle of cardiorenal deterioration. It is also associated with difficulties in the treatment of aggravating HF and chronic kidney disease (CKD) and, as a consequence, recurrent hospitalizations and death. As the worsening of renal function has an undeniably negative impact on the outcomes in patients with HF, searching for new treatment strategies and identification of biomarkers is necessary. This review is focused on the pathomechanisms in chronic kidney disease in patients with HF and therapeutic strategies for co-existing CKD and HF.

## 1. Introduction

Heart failure (HF) is a heterogeneous clinical syndrome resulting from injury and cardiac overload that in consequence leads to the elevation of intracardiac pressure and inadequate cardiac output. Due to left ventricular ejection fraction (LVEF), HF is divided into three main categories: HF with preserved (HFpEF; LVEF ≥ 50%), mildly reduced (HFmrEF; LVEF 41–49%), and reduced ejection fraction (HFrEF; LVEF ≤ 40%) [1]. Nevertheless, right ventricle dysfunction can also result in HF.

Patients suffering from HF are at high risk of comorbidities which are strictly connected with higher mortality risk, increased burden of healthcare costs, and adverse outcomes [2,3]. Additional chronic conditions are a major concern in heart failure. According to Chamberlain’s research [3], most heart failure patients have at least two chronic conditions. Furthermore, patients with HFpEF present an increased number of comorbidities compared to patients with HFrEF.

The heart and kidney are closely related. Their role is to maintain salt–water homeostasis and normal blood pressure. Renal impairment and disturbance of salt and water excretion results in an increase in cardiac preload as well as afterload. Furthermore, low cardiac output can decrease kidney perfusion and lead to kidney failure. Therefore, renal impairment is one of the most serious consequences of HF [4]. Primary pathomechanisms of this process are mainly reduced renal perfusion and venous congestion [5]. However, coexisting renal failure can also be caused by diabetes, arterial hypertension, or ischemic kidney disease. 

A decrease in glomerular filtration rate (GFR) seems to be the most significant determinant of the overall progression of HF [6]. Damman et al. [7] have reported that nearly half of patients with heart failure suffer from chronic kidney disease (CKD). Similar results were obtained by McAlister et al.; about 50% of subjects with HF presented estimated glomerular filtration rate (eGFR) 60 mL/min [8]. According to Kottgen’s research [9] incidence of HF in patients with eGFR < 60 mL/min per 1.73 m^2^ was 18/1000 person-years. CKD appears more common in HFpEF, nevertheless, worse outcomes are mostly related to HFmrEF and HFrEF [10]. 

## 2. Pathomechanism of Chronic Kidney Disease in Course of Heart Failure

### 2.1. Bidirectional Interplay between the Heart and the Kidneys 

**Figure 1:**
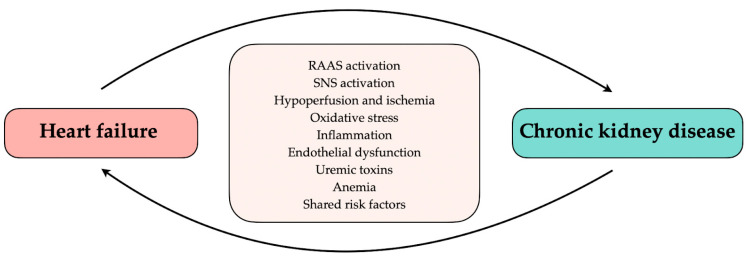
Relationship between heart failure and chronic kidney disease. RAAS, renin–angiotensin–aldosterone system; SNS, sympathetic nervous system.

The heart and kidneys play a major role in maintaining fluid homeostasis and normal blood pressure in the body. In physiological conditions, the cooperation between the heart and the kidneys enables a response to changes in renal perfusion, such as volume reduction or overload, which may cause ischemia or hyperperfusion injury [11]. On the other hand, sudden deterioration or chronic failure of one organ may be associated with decreased function of another. Renal salt and water excretion inability, improper renin secretion, and cardiomyopathic factors, such as myocardial infarction, left ventricular hypertrophy, and fibrosis, increase cardiac preload and afterload providing progressive volume and pressure overload [12,13]. Chronic existence of these abnormalities may lead to heart failure or aggravate an already existing HF. The common connection through the vascular bed, regulation by the sympathetic nervous system (SNS), and renin–angiotensin–aldosterone system (RAAS) cause stress on the renal nephrons. Further, insufficient kidney perfusion, due to low cardiac output, and renal venous congestion, caused by right heart failure, lead to kidney failure [12]. Furthermore, shared additional risk factors including diabetes mellitus, obesity, anemia, and iron deficiency or mineral disorder have an impact on the occurrence of HF in CKD and vice versa [13]. Common pathological mechanisms, shared risk factors, or systemic disorders may affect the heart and the kidneys, causing their simultaneous dysfunction (Figure 1). Moreover, studies imply elevated intra-abdominal pressure as a risk factor for kidney failure [14,15]. In addition, the interaction between cardiac disease and renal dysfunction leads to diuretic resistance [16]. As a result of considerable disorder overlap, the differentiation of which disease is primary and which is secondary may be complicated and challenging. This distinction may be needed to develop new treatments to improve renal and cardiac function.

The coexistence of abnormalities in the function or structure of both the heart and the kidneys, in which impairment of one organ leads to damage of the other, is usually termed cardiorenal syndrome (CRS) [4]. CRS is characterized based on which organ is primarily affected and whether it is acute or chronic damage [11]. One of the subtypes, CRS type 2, refers to persistent impairment of cardiac function as a source of potentially irreversible kidney damage and chronic kidney disease [11]. 

Approximately half (49%) of patients with heart failure suffer from CKD [7]. Even a small decrease in GFR is associated with increased mortality among patients with chronic heart failure (CHF) [17]. As the degree of chronic renal autoregulation following acute or chronic heart failure is still unclear, it might be a case for future study on type 2 CRS [18]. 

### 2.2. Neurohormonal Changes

Hypotension and hypoperfusion of the organs in the course of chronic heart failure lead to the activation of compensatory mechanisms, especially the stimulation of the renin–angiotensin–aldosterone system and the sympathetic system [19]. As a result of RAAS activation, angiotensin II (AII) stimulates the sympathetic nervous system, which innervates the afferent and efferent arteriole of the glomerulus. Activation of the sympathetic nervous system leads to the contraction of both vessels, which results in a decrease of renal blood flow and GFR. The direct effect of AII on the cardiovascular system is to increase preload and afterload of the heart (through increased sodium and water retention in the kidneys), resulting in increased myocardial oxygen demand [20]. AII also elevates the level of arginine vasopressin (AVP) which contributes to the progression of CKD [21]. 

Studies have shown a significant relationship between increased N-terminal pro-brain natriuretic peptide (NT-proBNP) and decreased GFR in patients with CHF [22,23]. Natriuretic peptides can be elevated among others because of the low elimination of the molecules by the injured kidneys. Increased levels of BNP and NT-proBNP may signal an elevated risk for accelerated progression of CKD to ESRD [22]. B-type natriuretic peptide (BNP) may be an additional marker to detect the involvement of kidneys in ventricular stress [24].

However, the role of NT-pro BNP in CHF with CKD is still not studied enough. To elucidate the benefits of this marker additional research is crucial to better understand the cause-and-effect relationship.

### 2.3. Inflammation

Induced by CHF, hypotension and organ hypoperfusion result in increased production of AVP and endothelin (EI) stimulated by ATII [15,19]. EI is a pro-inflammatory, pro-fibrotic, and vasoconstrictor protein. It stimulates transforming growth factor β (TGF-β) and transcription nuclear factor κB (NF-κB), maintaining inflammation in the kidney. Moreover, aldosterone stimulates the production of fibronectin, leading to the intensification of fibrosis processes in the glomeruli [25]. Besides the kidneys, angiotensin II type 1 receptors are also found in the heart. The release of TGF-β1 and ETI from the cardiac fibroblasts results in cardiac hypertrophy [15]. It is suggested that the promotion of inflammation by the RAAS may be the main mechanism impairing renal function in patients with CHF and left ventricular systolic function [26]. Elevated plasma levels of C-reactive protein and interleukin 6 (IL-6) correlate with left ventricular hypertrophy (LVH) and contractile dysfunction in CKD patients [27]. 

In a study by Freise et al., inflammatory processes mediated by tumor necrosis factor (TNF) and interleukin 10 (IL-10) have an impact on pathobiological responses in the arteries of children with CKD and thus are associated with tissue remodeling and cardiovascular disease [28]. Research on the inhibition of pro-inflammatory interleukin IL-1β in patients with renal disorders has promising prospects [29].

### 2.4. Oxidative Stress

Persistent hypoperfusion and hypoxia in CHF exacerbate oxidative stress in the kidney. Moreover, caused by AII activation of the oxidases, reduced nicotinamide adenine dinucleotide (NADH) and nicotinamide adenine dinucleotide phosphate (NADPH) lead to the production of reactive oxygen species (ROS), which together with the impaired antioxidant barrier also cause oxidative stress [29,30]. The consequence is a decrease in the bioavailability of nitric oxide (NO) and endothelial dysfunction [30,31]. It has been reported that chronic kidney disease, inducing damage to endothelial cells, promotes atherosclerosis and coronary artery disease (CAD). Albuminuria may also be associated with endothelial dysfunction [29]. The level of the oxidative stress marker, 8-isoprostane, increases with the progression of CKD [32].

Oxidative stress also enhances the inflammatory response by activating phagocytes [33] and increasing the production of pro-inflammatory cytokines [34], especially interleukin 1 (IL-1), IL-6, and tumor necrosis factor α (TNF-α). It results in the persistence of chronic inflammation, leading to progressive loss of kidney function through toxic damage [35]. Elevated levels of pro-inflammatory cytokines C-reactive protein (CRP), IL-6, and TNF have been shown to be associated with the risk of myocardial infarction and mortality [36].

### 2.5. Uremic Toxins

Uremic toxins, retained through loss of renal excretion, have been described as contributing factors to the development of cardiac remodeling and kidney damage, but their exact role is less understood. It is suggested that uremic toxins start accumulating from the early stage of CKD [37]. Some works suggest direct cardiotoxicity of some toxins, such as indoxyl sulfate and p-cresyl sulfate [38,39]. Indoxyl sulfate may have cytotoxic effects on endothelial cells in patients with end-stage renal disease (ESRD) [40]. It also generates ROS and inducts fibrosis and inflammation [39]. 

However, the most crucial toxins are asymmetric dimethylarginine (ADMA), advanced glycation endproducts (AGE), and trimethyl amine N-oxide (TMAO) [30]. ADMA toxin participates in the regulation of NO, directly reducing the intraepithelial phosphorylation of NO synthase. It also regulates the production of ROS, activates the RAAS, and is associated with renal anemia. AGE do not degrade and, accumulating in the heart and kidneys, lead to tissue damage. On the other hand, TMAO promotes fibrosis, and its increased level is associated with the atherosclerotic process [39] and poor prognosis in patients with HF [37,41]. Moreover, it has been reported that urinary TMAO was significantly lower in vegetarian and vegan subjects compared to omnivorous subjects, which remains a point of interest [39] and a starting point in developing a therapeutic strategy.

Due to the fact that the cardio and nephrotoxicity of uremic toxins is very high, finding an appropriate strategy that will prevent its accumulation in the body is necessary. Uremic toxins should be considered in the treatment of cardio-renal syndrome.

### 2.6. Anemia

In recent years, researchers have dwelt on anemia as a crucial factor in the pathophysiology and progression of heart failure and chronic kidney disease. The incidence of anemia increases with CKD and HF deterioration [42]. The etiology of anemia in patients with HF is multifactorial. The release of antioxidants from damaged by ischemia red blood cells (RBC) may generate oxidative stress [43]. Excessive fluid retention combined with gastrointestinal disorder results in malabsorption and iron deficiency. Released in CKD and cardiovascular disease (CVD), proinflammatory cytokines increase hepcidin-25 concentrations, also leading to iron deficiency by decreasing the absorption of intestinal iron. This also inhibits iron release from internal stores which causes impaired Hb synthesis and iron-restricted erythropoiesis [44]. Erythropoietin (EPO) deficiency in CKD is significant [45]. As kidney function declines during heart failure, the mechanism of EPO production as a response to renal hypoxia becomes impaired [46,47]. This results in insufficient oxygen delivery and tissue hypoxia, which leads to peripheral vasodilation and stimulation of SNS and RAAS activity. Furthermore, it releases AVP resulting in vasoconstriction, salt and water retention, and chronic renal venous congestion [15]. In consequence, renal perfusion decreases and cardiac burden increases [44], leading to progressive nephron loss and renal fibrosis. Chronic anemia results in LVH and myocardial cell death as a consequence of ischemia and necrosis [15].

Drüeke et al. [48] reported that early complete correction of anemia in patients with CKD does not reduce cardiovascular risk. Another study suggests that intravenous iron administration reduces symptoms in patients with heart failure and CKD in stage 3. Moreover, high doses of iron may reduce the number of hospitalizations for HF in patients receiving dialysis by 44% [49].

## 3. Treatment

The European guidelines recommend four well-known drugs as a gold standard in the treatment of chronic heart failure [1]. They include (1) renin–angiotensin–aldosterone system inhibitor (RAAS-I; ACEI as a first choice), (2) beta-blocker, (3) mineralocorticoid receptor antagonists (MRAs), and (4) sodium–glucose cotransporter 2 (SGLT2) inhibitor. All are presented in Table 1. Additionally, a large role for diuretics is also indicated. Meta-analysis on 95,444 patients with heart failure with reduced ejection fraction (HFrEF) revealed that combination therapy with these four drugs resulted in a 61% lower risk of death compared to not using all of these recommended medications [50]. Vaduganathan et al. [51] estimated that this comprehensive therapy can extend patients’ life by 8.3 years for those who are 55 years old and 2.7 years for people at the age of 80. This is a significant finding, particularly for patients with HF who also suffer from others comorbidities. Accrual of comorbidity is strictly associated with a notable increase in excess loss of life, especially for CKD [52].

### 3.1. Renin–Angiotensin–Aldosterone System Inhibitors

The RAAS plays a principal role in the development and progression of heart failure. Its main functions are the regulation of blood pressure and the maintenance of water and electrolyte balance in the body. Angiotensin-converting enzyme inhibitors (ACEI) significantly reduce mortality and hospitalizations as well as slow disease progression [57]. They are effective in all degrees of severity of HF including left ventricular dysfunction. The other drugs which also manipulate the RAAS are angiotensin receptor blockers (ARB). 

Bowling et al. [58] revealed that enalapril (ACEI) is a promising drug for patients with HFrEF and CKD due to its properties that reduce mortality and hospitalization. Studies show that ACEI should be used in all patients with HFrEF [55]. Furthermore, intake of ARB is associated with a reduction in all-cause mortality in patients with acute HF and CKD [59]. However, this kind of treatment requires consistent monitoring of creatinine and potassium. Furthermore, in stages 4 and 5 CKD dose modification may be indispensable [53]. As is well known, higher doses of ACEI can cause hyperkalemia in dialysis patients. Increased potassium level is an infrequent side effect; nevertheless, it increases with worsening kidney function [60].

CKD significantly impedes pharmacologic therapy in heart failure. Sacubitril/valsartan, an angiotensin receptor–neprilysin inhibitor (ARNI), might be the solution for HFrEF patients. Sacubitril inhibits neprilysin, increasing diuresis, natriuresis, and vasodilatation and suppresses adverse cardiac remodeling [61]. Neprilysin inhibition is suggested to contribute to the preservation of renal function by enhancing the bioavailability of renal natriuretic peptides. Not only does it counteract the side effects of over-activation of the RAAS, but it also decreases the risk of cardiovascular mortality [59]. Additionally, new recommendations indicate that ACEI and ARB should be replaced by ARNI in patients in patients with New York Heart Association class II or III HFrEF [62]. It is worth emphasizing that despite the additional renal benefits, sacubitril/valsartan has not been extensively researched in patients with advanced stages of CKD. 

### 3.2. Beta-Blockers

Beta-blockers have the ability to improve hemodynamics in chronic heart failure. Their role in treatment is equally important as ACEI [63]. Initiation of therapy with beta-blocker is as safe and efficacious as with enalapril. It has been proven that metoprolol administered in addition to standard therapy lower mortality in patients with HFrEF [64]. Furthermore, carvedilol has been reported to reduce the number of hospitalizations as well as listing for cardiac transplantation [65]. In regards to the clinical picture, in the study Metoprolol in Dilated Cardiomyopathy (MDC) patients noted an improvement in symptom score [66]. 

The sympathetic nervous system has a huge impact on renal function. Its overactivity is specific to CKD. Modulation of beta1 receptors is associated with cardiac output and renin release; in turn, beta2 receptors are responsible for renovascular dilation. 

Besides all the benefits of beta-blockers, their advantages in the dialysis population can be uncertain due to their heterogeneity. It is worth noting that greater use of this kind of therapy could contribute to a decline in the risk of heart failure, which is the most common cause of death in the first year of starting dialysis. Zhou et al. [54] demonstrated a significant decrease in the mortality rate among hemodialysis subjects with HF. Even so, the same effects were not noted in the first 6 months of dialysis.

### 3.3. Mineralocorticoid Receptor Antagonists

Another noteworthy therapeutic option are MRAs. They work by blocking receptors that bind aldosterone. Activation of the mineralocorticoid receptors has been proven to result in negative consequences among people with cardiovascular disease. For example, it contributes to cardiac fibrosis [67], and as it is well known the key pathogenetic element of HF is myocardial remodeling.

Spironolactone or eplerenone is recommended in all patients with HFrEF in addition to standard therapy [1]. They are suggested for more comprehensive inhibition of the RAA. Studies have shown that extending HF therapy with spironolactone improves mortality outcomes in hemodialysis patients [68]. Zannad et al. demonstrated that eplerenone is an effective drug in reducing mortality and hospitalization among patients with systolic HF who present mild symptoms [69]. Moreover, the blockade of aldosterone receptors can also contribute to a decline in morbidity and death among people who suffer from severe HF [70]. Nevertheless, it is worth remembering their potential negative consequences, such as hyperkalemia or GFR reduction. Fortunately, it is emphasized that these adverse effects are not common—although close monitoring and careful uptitration of dosage should be applied.

### 3.4. Sodium–Glucose Cotransporter 2 Inhibitors

SGLT2 inhibitors are one of the most recent unique groups of drugs. They are commonly used in patients with type 2 diabetes as a glucose-lowering therapy. Their mechanism is based on the inhibition of renal glucose reuptake. Its additional benefits include weight loss, improvement of insulin tissue sensitivity, or blood pressure reduction. According to 2021 ESC Guidelines, SGLT2 inhibitors such as canagliflozin, dapagliflozin, empagliflozin, ertugliflozin, and sotagliflozin are recommended in patients with HF regardless of diabetes status [1]. The suggested starting and target dose for empagliflozin/dapagliflozin is 10 mg once daily [1].

Zannad et al. concluded that both empagliflozin and dapagliflozin have a wide range of positive effects in HFrEF therapy. They reduce cardiovascular and all-cause death, and decrease the number of hospitalizations [71]. Furthermore, it has been shown that dapagliflozin treatment is also very effective at both primary and secondary prevention of HF [72]. Additionally, it alleviates HF symptoms and improves general health status [73]. Similar results were obtained with empagliflozin in EMPA-REG OUTCOME where a reduction in cardiovascular mortality was noted [74].

SGLT2 inhibitors are also characterized by renoprotective effects. For example, canagliflozin has been proven to reduce albuminuria as well as the risk of sustained loss of kidney function. As stated in the research by Heerspink et al., the risk of a composite of a sustained decline in the GFR of at least 50% was remarkably lower in the dapagliflozin group compared to the placebo group [75]. In the DECLARE-TIMI 58 study, dapagliflozin presented a favorable effect on the urinary albumin-to-creatinine ratio (UACR) [76]. Moreover, McMurray et al. demonstrated that it also decreases the risk of kidney failure and prolongs survival in CKD [72]. What is clinically important due to their diuretic properties they also can contribute to a reduction in loop diuretic requirement [77].

Besides all these benefits, it is worth remembering that SGLT2 inhibitors, like every drug, have adverse effects. Recurrent genital fungal infections and volume depletion-related issues are some of them. Fortunately, they are generally mild and easy to treat [56]. After initiation of SGLT2 inhibitors, a slight decrease in eGFR can be observed; however, it is not alarming and should not be a reason for treatment discontinuation.

### 3.5. Medical Devices

Devices such as cardiac resynchronization therapy (CRT) and implantable cardioverter-defibrillator (ICD) have become novel approaches for HF patients. CRT is currently a common nonpharmacologic therapy in patients with HF. ICD is nowadays the gold standard in the prevention of arrhythmic sudden cardiac death (SCD) episodes, and as it is widely known, ventricular arrhythmias are one of the major causes of death in HF patients. 

Factors that determine the validity of devices in the treatment of HF co-occurring with CKD are patients’ age, bloodstream infection, or rate of CKD progression. Even though these new therapeutic strategies have been widely investigated in major randomized controlled studies, there is still insufficient evidence for the appropriateness of CRT/ICD implementation in patients with HFmrEF [1].

Tang et al. [78] demonstrated that the addition of CRT to an ICD reduced mortality and hospitalization for HF. What is important, more than half of patients had eGFR < 60 mL/min per 1.73 m^2^. However, the ICD-CRT combination was associated with more adverse events than the ICD group. Another study revealed that mortality rates were high in a population undergoing dialysis, despite the use of implantable cardioverter-defibrillators [79]. Additionally, post-implantation infections were a significant issue among those subjects, especially in the first year after implantation. Diabetics and people who previously had an infection were particularly vulnerable. Furthermore, Jukema et al. concluded that in dialysis patients, prophylactic ICD therapy did not result in a mortality decrease, nor SCD [80]. 

### 3.6. Kidney Replacement

Careful consideration of all advantages and disadvantages is essential when starting kidney replacement therapy (KRT). eGFR <20 mL/min per 1.73 m^2^ is an appropriate moment for a decision on whether KRT should be implemented. Co-occurring conditions, patient expectations, and life quality should be taken into account.

Banerjee et al. [81] showed that dialysis patients with comorbidities such as congestive HF or pulmonary edema had very poor survival. Five-year survival for HF was 12.5%. However, it is worth mentioning that those subjects were older compared with patients who presented congestive HF without kidney failure. Furthermore, the main causes of CKD were diabetes mellitus or hypertension. However, an important issue is the side effects of arteriovenous fistula or graft for hemodialysis. Reddy et al. [82] revealed that they may lead to dilatation of the left atrium and right ventricle and related HF. 

In another study, continuous ambulatory peritoneal dialysis (CAPD) was assessed [83]. The results showed that in advanced HF, the use of CAPD had a positive impact on parameters such as Minnesota Living With Heart Failure Questionnaire (MLWHFQ) and NYHA class. The rate of adverse events was acceptable. Koch et al. obtained similar results [84]. They confirmed peritoneal dialysis (PD) as a safe, efficient, and well-tolerated therapeutic option for people with HF and CKD. Additionally, PD was associated with a notable reduction in hospitalization days and an improvement in left ventricular ejection fraction [85]. 

### 3.7. Heart Failure with Preserved Ejection Fraction

Approximately half of HF patients have a preserved ejection fraction, and the prevalence is still growing. As mentioned before, CKD is more frequent among patients with HFpEF. Nevertheless, there is currently no reliable research that has indicated effective therapy for HFpEF. Drugs such as RAAS-I, MRAs, or SGLT2 inhibitors are well tolerated and can decrease the risk of hospitalization [86]. However, there is no compelling evidence for the reduction of mortality and morbidity. 

Treatment in HFpEF includes symptom management with diuretics and optimization of comorbidities. The 2021 ESC Guidelines highlight the role of diuretics in congested patients [1]. They reduce the risk of HF aggravation and improve exercise capacity [87]. Additionally, they are also crucial for controlling symptoms of volume overload. 

It is worth remembering that nonsteroidal anti-inflammatory drugs can lead to fluid retention in CKD patients, therefore they should not be used frequently.

## 4. Future Therapeutic Strategies

### 4.1. SGLT2 Inhibitors

Recent clinical trials about SGLT2 inhibitors have shown that flozin therapy, especially with dapagliflozin, in patients with heart failure reduces mortality and hospitalizations [55,75], regardless of the presence of diabetes mellitus [88], GFR rate, or albuminuria. Furthermore, SGLT2 inhibition is associated with a decreased progression of CKD in patients with and without diabetes [75]. Empagliflozin reduces eGFR decline, including in patients with eGFR as low as 20 mL/min per 1.73 m^2^ [89]. Moreover, SGLT2 inhibitors reduce oxidative stress, which may be valuable regarding vascular alterations in patients with or without CKD [90].

### 4.2. Finerenone

The novel nonsteroidal MRA finerenone has a beneficial effect on cardiovascular and kidney organ protection, particularly in patients with albuminuria and type 2 diabetes. The comparative analysis of the effect of SGLT2 inhibitor canagliflozin versus finerenone on cardiorenal outcomes demonstrated cardiorenal benefits of a similar magnitude [91]. 

Research on finerenone demonstrates the benefits of therapy in patients with HF across CKD stages [92]. Finerenone significantly reduced the risk of hospitalizations or cardiovascular death in CKD patients, regardless of eGFR or UACR at baseline. Bakris et al. [93] also have shown the effect of finerenone therapy on kidney outcomes. In patients with CKD and diabetes mellitus type 2, treatment with finerenone resulted in lower risks of CKD progression or death from the renal cause, and a lower risk of cardiovascular events.

Despite many benefits of finerenone in patients suffering from HF and CKD, this treatment may often lead to hyperkalemia complications, which are critical in patients with, e.g., diabetic nephropathy or demanding hemodialysis.

### 4.3. Canakinumab

Randomized controlled trial have shown that canakinumab therapy may be associated with a reduction in cardiovascular events, also in post-myocardial infarction patients with CKD [94]. Nevertheless, the data from this research have not provided strong evidence for renal protection in other groups than in patients with moderate CKD group [95]. To clarify the potential for kidney protection with canakinumab further research in that direction is recommended.

## 5. Conclusions

Concomitant diseases are a common and unfavorable indicator for people with HF. They are closely related to higher mortality and increased hospitalization. In this review, we focused on the important therapeutic strategies for co-existing CKD and HF. We paid attention to both pharmacological and non-pharmacological treatment. We drew attention to the main groups of drugs mentioned in the 2021 ESC Guidelines and considered the advantages and disadvantages of devices such as ICDs and CRTs. Moreover, we broached the subject of kidney transplantation. These findings may shed novel insight into the underlying mechanisms and identify potential therapeutic targets for the management of HF patients and multimorbidity in the future.

## Figures and Tables

**Table 1:** Pharmacotherapy of HFrEF in patients with CKD.

Agents	Recommendations	Comments	References
Renin–angiotensin–aldosterone system inhibitors	Should be used in all patients	Creatinine and potassium monitoring is necessary CKD Stages 4 and 5: dose modification may be needed	[1,53]
β-Blockers	Should be used in all patients	Uncertain effects in dialysis population	[1,54]
Mineralocorticoid receptor antagonists	Should be used in all patients	Creatinine and potassium monitoring is necessary	[1,55]
Sodium–glucose cotransporter 2 inhibitors	Can be used in patients with/without diabetes	Risk of recurrent genital fungal infections and hypotension	[1,56]

## Data Availability

The data used in this article are sourced from materials mentioned in the References section.

## Abbreviations

AII	angiotensin II
ACEI	angiotensin-converting enzyme inhibitors
ADMA	asymmetric dimethylarginine
AGE	advanced glycation endproducts
ARB	angiotensin receptor blockers
AVP	arginine vasopressin
BNP	B-type natriuretic peptide
CAD	coronary artery disease
CAPD	continuous ambulatory peritoneal dialysis
CHF	chronic heart failure
CKD	chronic kidney disease
CRP	C-reactive protein
CRS	cardiorenal syndrome
CRT	cardiac resynchronization therapy
CVD	cardiovascular disease
EI	endothelin
eGFR	estimated glomerular filtration rate
EPO	erythropoietin
ESA	erythropoiesis-stimulating agents
ESRD	end-stage renal disease
GFR	glomerular filtration rate
HF	heart failure
HFmrEF	heart failure with mildly reduced ejection fraction
HFpEF	heart failure with preserved ejection fraction
HFrEF	heart failure with reduced ejection fraction
Il-1	interleukin 1
Il-6	interleukin 6
KRT	kidney replacement therapy
LVEF	left ventricular ejection fraction
LVH	left ventricular hypertrophy
MLWHFQ	Minnesota Living With Heart Failure Questionnaire
MRAs	mineralocorticoid receptor antagonists
NADH	nicotinamide adenine dinucleotide
NADPH	nicotinamide adenine dinucleotide phosphate
NF-κB	transcription nuclear factor κB
NO	nitric oxide
NT-proBNP	N-terminal pro-brain natriuretic peptide
NYHA	New York Heart Association
PD	peritoneal dialysis
RAA	renin–angiotensin–aldosterone
RAAS	renin–angiotensin–aldosterone system
RAAS-I	renin–angiotensin–aldosterone system inhibitors
RBC	red blood cells
ROS	reactive oxygen species
SCD	sudden cardiac death
SGLT2	sodium–glucose cotransporter 2
SNS	sympathetic nervous system
TGF-β	transforming growth factor β
TMAO	trimethyl amine N-oxide
TNF-α	tumor necrosis factor α
UACR	urinary albumin-to-creatinine ratio
